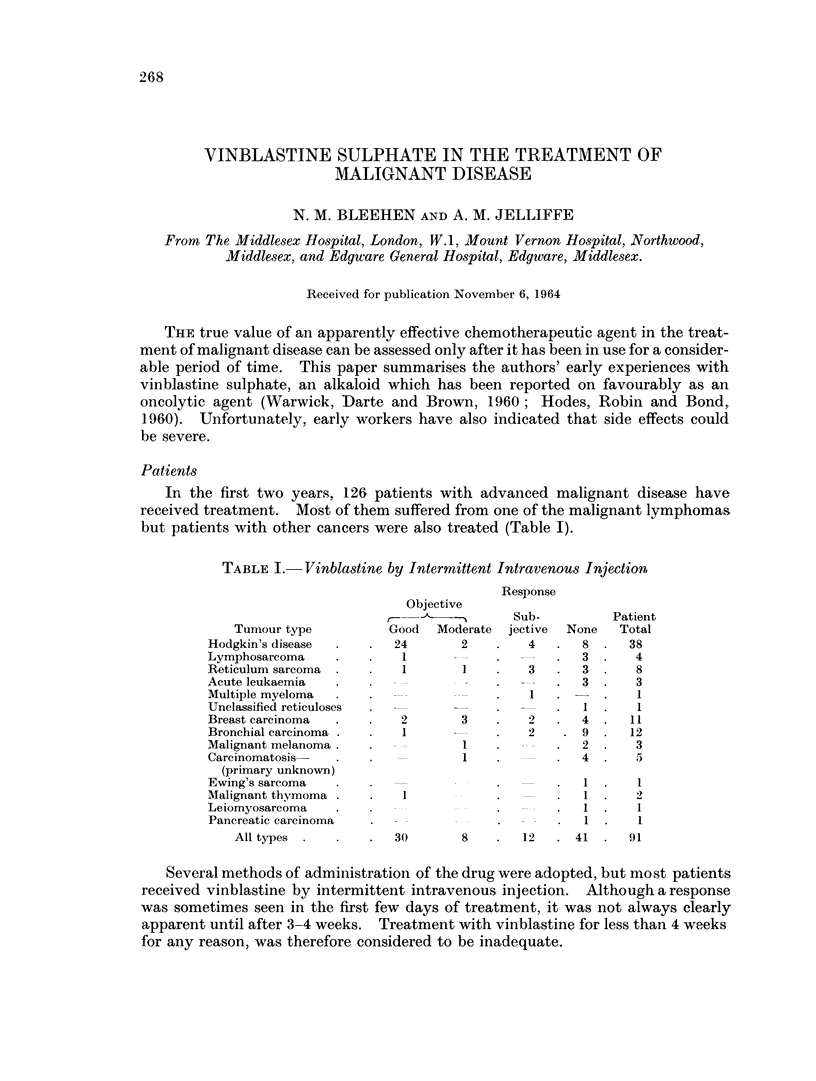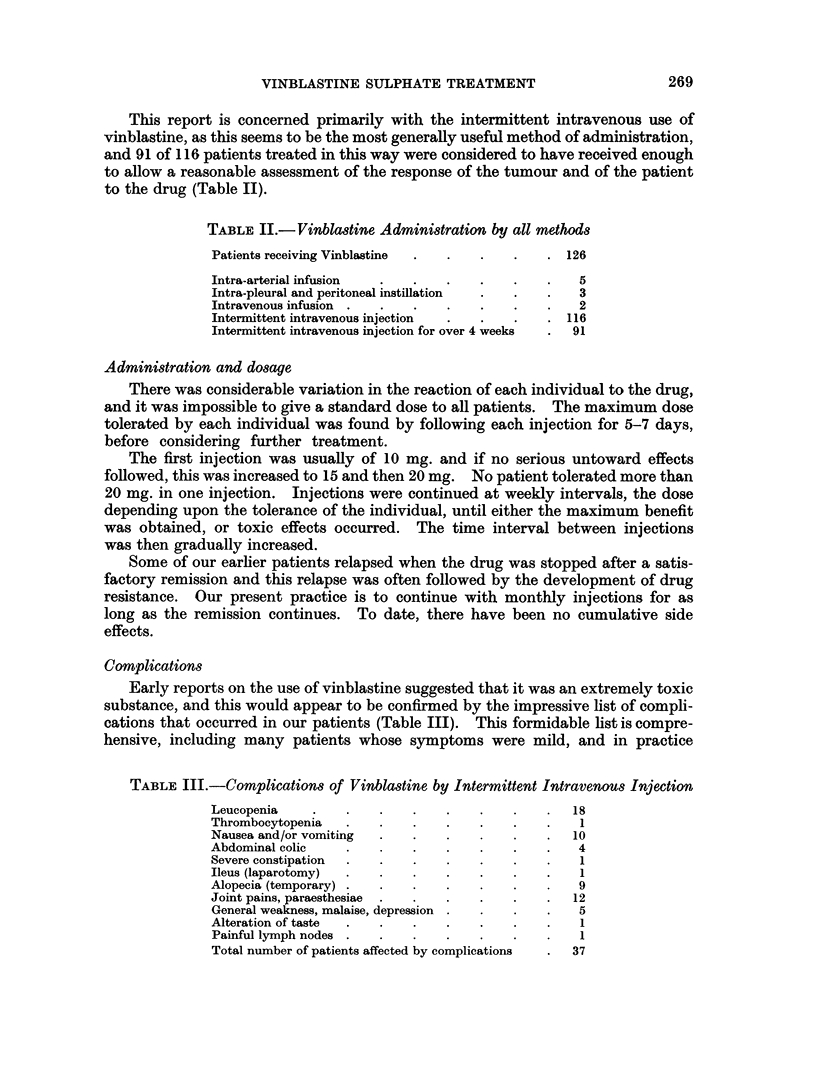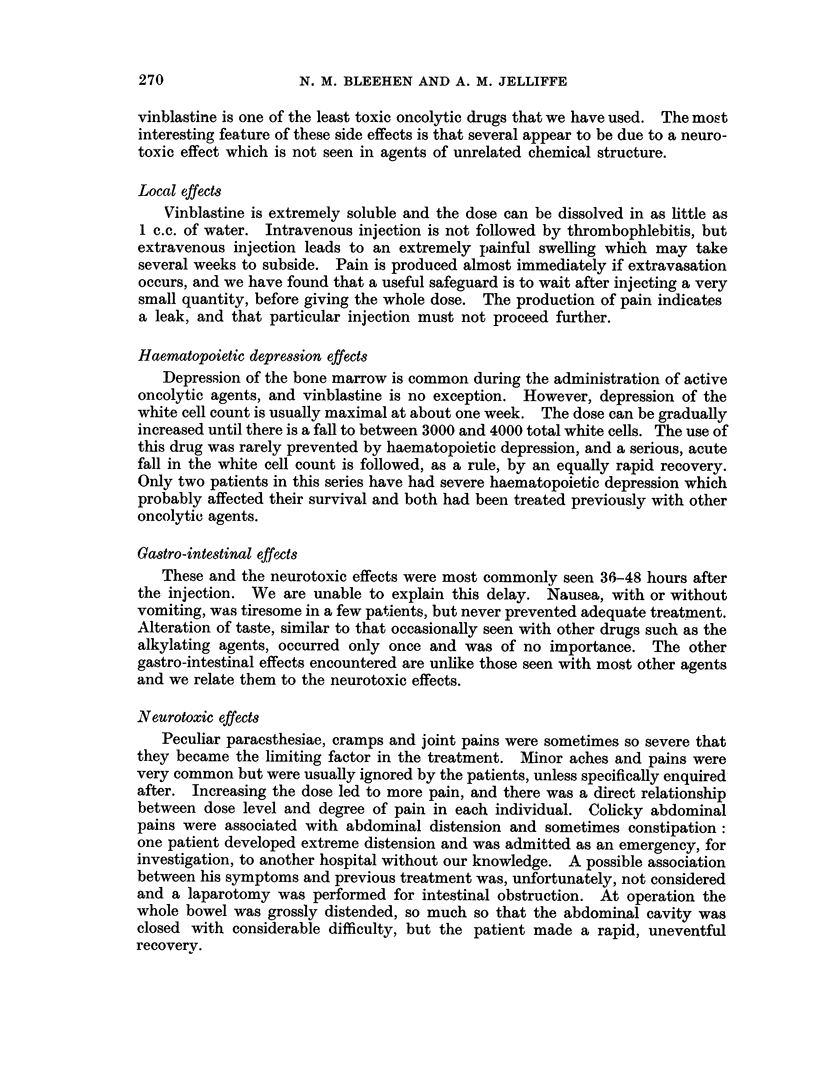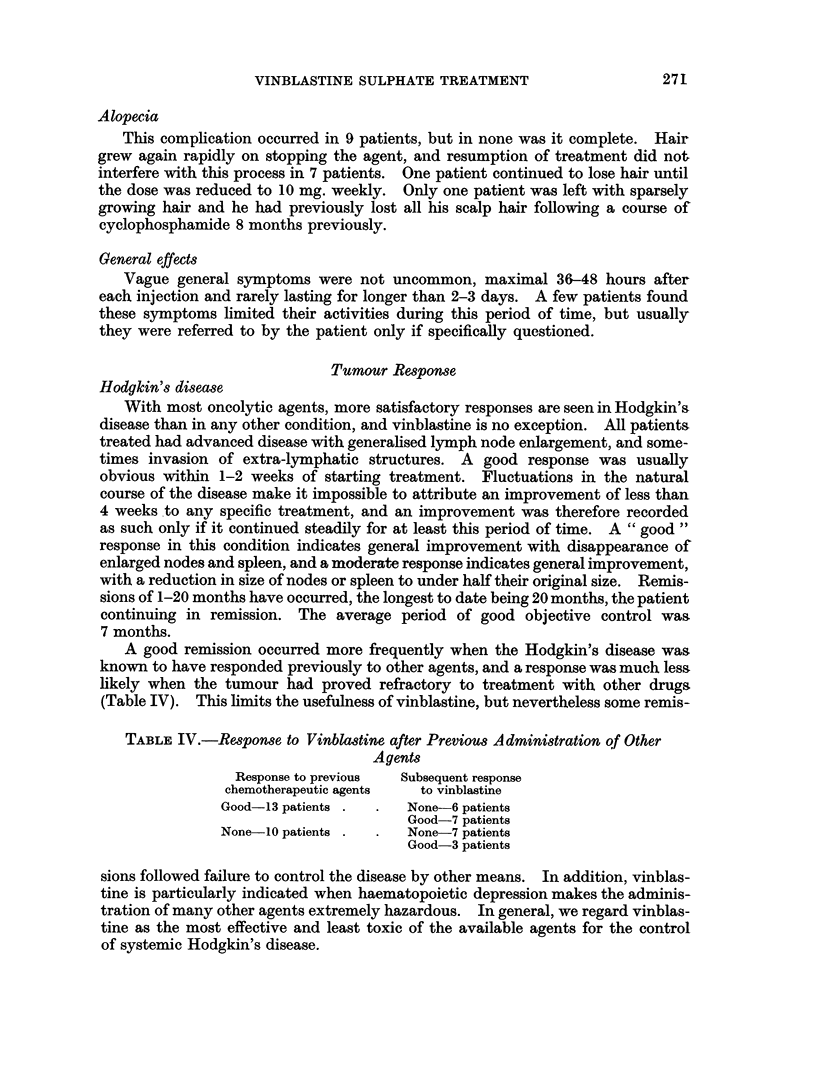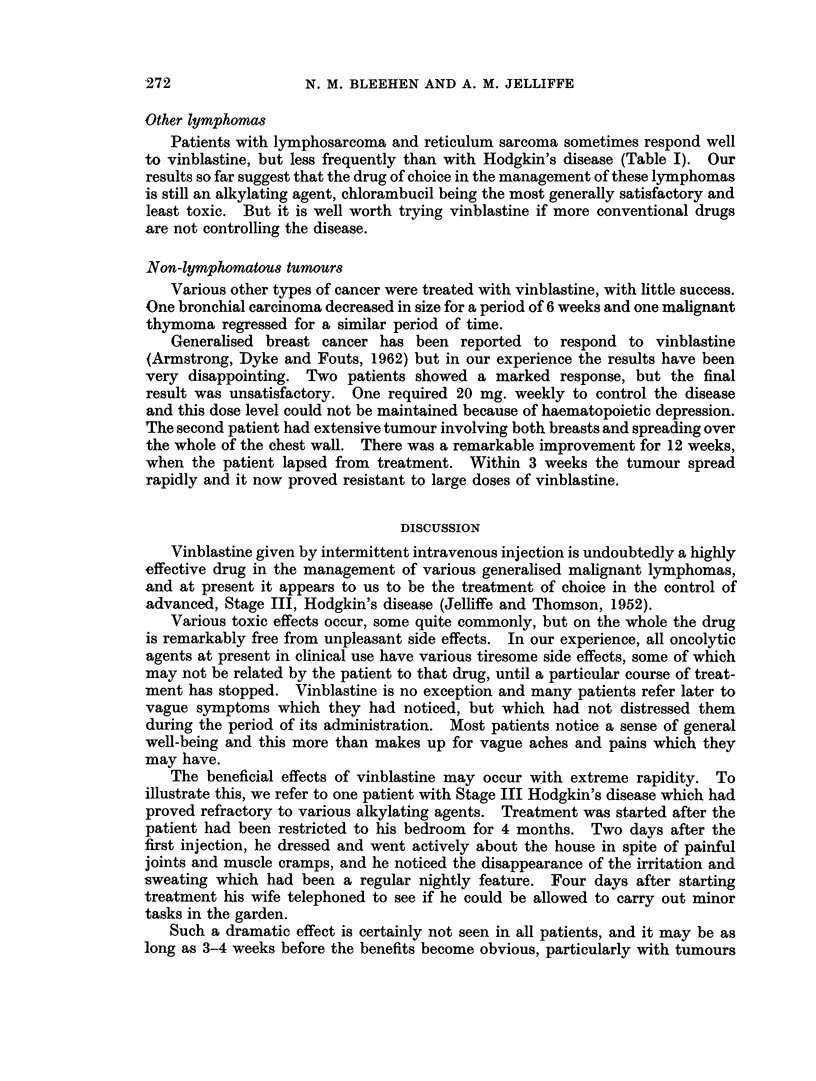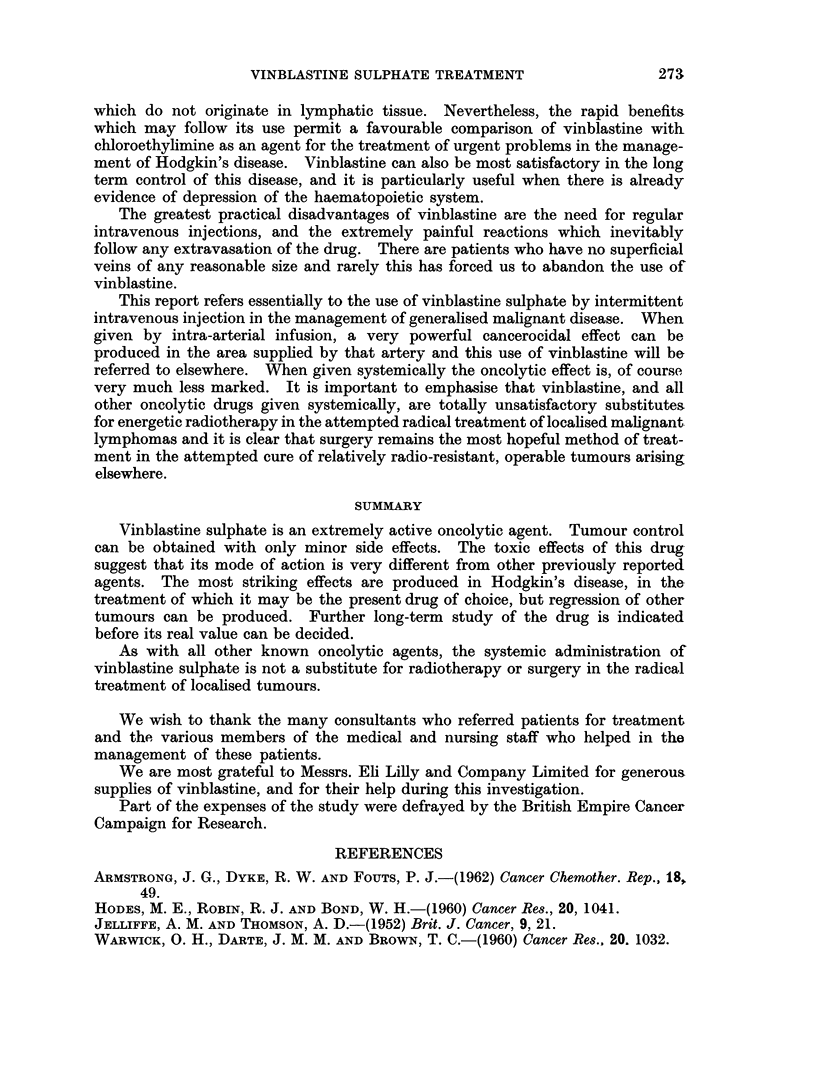# Vinblastine Sulphate in the Treatment of Malignant Disease

**DOI:** 10.1038/bjc.1965.30

**Published:** 1965-06

**Authors:** N. M. Bleehen, A. M. Jelliffe


### VINBLASTINE SULPHATE IN THE TREATMENT OF MALIGNANT DISEASE

N. M. BLEEHEN AND A. M. JELLIFFE

*From The Middlesex Hospital, London, W.1, Mount Vernon Hospital, Northwood, Middlesex, and Edgware General Hospital, Edgware, Middlesex.*

Received for publication November 6, 1964

THE true value of an apparently effective chemotherapeutic agent in the treatment of malignant disease can be assessed only after it has been in use for a considerable period of time. This paper summarises the authors' early experiences with vinblastine sulphate, an alkaloid which has been reported on favourably as an oncolytic agent (Warwick, Darte and Brown, 1960 ; Hodes, Robin and Bond, 1960). Unfortunately, early workers have also indicated that side effects could be severe.

*Patients*

In the first two years, 126 patients with advanced malignant disease have received treatment. Most of them suffered from one of the malignant lymphomas but patients with other cancers were also treated (Table I).

TABLE I.—*Vinblastine by Intermittent Intravenous Injection*

| | Response | | | | |
|---|---|---|---|---|---|
| | Objective | | | | |
| Tumour type | Good | Moderate | Subjective | None | Patient Total |
| Hodgkin's disease | 24 | 2 | 4 | 8 | 38 |
| Lymphosarcoma | 1 | — | — | 3 | 4 |
| Reticulum sarcoma | 1 | 1 | 3 | 3 | 8 |
| Acute leukaemia | — | — | — | 3 | 3 |
| Multiple myeloma | — | — | 1 | — | 1 |
| Unclassified reticuloses | — | — | — | 1 | 1 |
| Breast carcinoma | 2 | 3 | 2 | 4 | 11 |
| Bronchial carcinoma | 1 | — | 2 | 9 | 12 |
| Malignant melanoma | — | 1 | — | 2 | 3 |
| Carcinomatosis—(primary unknown) | — | 1 | — | 4 | 5 |
| Ewing's sarcoma | — | — | — | 1 | 1 |
| Malignant thymoma | 1 | — | — | 1 | 2 |
| Leiomyosarcoma | — | — | — | 1 | 1 |
| Pancreatic carcinoma | — | — | — | 1 | 1 |
| All types | 30 | 8 | 12 | 41 | 91 |

Several methods of administration of the drug were adopted, but most patients received vinblastine by intermittent intravenous injection. Although a response was sometimes seen in the first few days of treatment, it was not always clearly apparent until after 3–4 weeks. Treatment with vinblastine for less than 4 weeks for any reason, was therefore considered to be inadequate.

This report is concerned primarily with the intermittent intravenous use of vinblastine, as this seems to be the most generally useful method of administration, and 91 of 116 patients treated in this way were considered to have received enough to allow a reasonable assessment of the response of the tumour and of the patient to the drug (Table II).

TABLE II.—*Vinblastine Administration by all methods*

| | |
|---|---|
| Patients receiving Vinblastine | 126 |
| Intra-arterial infusion | 5 |
| Intra-pleural and peritoneal instillation | 3 |
| Intravenous infusion | 2 |
| Intermittent intravenous injection | 116 |
| Intermittent intravenous injection for over 4 weeks | 91 |

*Administration and dosage*

There was considerable variation in the reaction of each individual to the drug, and it was impossible to give a standard dose to all patients. The maximum dose tolerated by each individual was found by following each injection for 5–7 days, before considering further treatment.

The first injection was usually of 10 mg. and if no serious untoward effects followed, this was increased to 15 and then 20 mg. No patient tolerated more than 20 mg. in one injection. Injections were continued at weekly intervals, the dose depending upon the tolerance of the individual, until either the maximum benefit was obtained, or toxic effects occurred. The time interval between injections was then gradually increased.

Some of our earlier patients relapsed when the drug was stopped after a satisfactory remission and this relapse was often followed by the development of drug resistance. Our present practice is to continue with monthly injections for as long as the remission continues. To date, there have been no cumulative side effects.

*Complications*

Early reports on the use of vinblastine suggested that it was an extremely toxic substance, and this would appear to be confirmed by the impressive list of complications that occurred in our patients (Table III). This formidable list is comprehensive, including many patients whose symptoms were mild, and in practice

TABLE III.—*Complications of Vinblastine by Intermittent Intravenous Injection*

| | |
|---|---|
| Leucopenia | 18 |
| Thrombocytopenia | 1 |
| Nausea and/or vomiting | 10 |
| Abdominal colic | 4 |
| Severe constipation | 1 |
| Ileus (laparotomy) | 1 |
| Alopecia (temporary) | 9 |
| Joint pains, paraesthesiae | 12 |
| General weakness, malaise, depression | 5 |
| Alteration of taste | 1 |
| Painful lymph nodes | 1 |
| Total number of patients affected by complications | 37 |

vinblastine is one of the least toxic oncolytic drugs that we have used. The most interesting feature of these side effects is that several appear to be due to a neurotoxic effect which is not seen in agents of unrelated chemical structure.

*Local effects*

Vinblastine is extremely soluble and the dose can be dissolved in as little as 1 c.c. of water. Intravenous injection is not followed by thrombophlebitis, but extravenous injection leads to an extremely painful swelling which may take several weeks to subside. Pain is produced almost immediately if extravasation occurs, and we have found that a useful safeguard is to wait after injecting a very small quantity, before giving the whole dose. The production of pain indicates a leak, and that particular injection must not proceed further.

*Haematopoietic depression effects*

Depression of the bone marrow is common during the administration of active oncolytic agents, and vinblastine is no exception. However, depression of the white cell count is usually maximal at about one week. The dose can be gradually increased until there is a fall to between 3000 and 4000 total white cells. The use of this drug was rarely prevented by haematopoietic depression, and a serious, acute fall in the white cell count is followed, as a rule, by an equally rapid recovery. Only two patients in this series have had severe haematopoietic depression which probably affected their survival and both had been treated previously with other oncolytic agents.

*Gastro-intestinal effects*

These and the neurotoxic effects were most commonly seen 36–48 hours after the injection. We are unable to explain this delay. Nausea, with or without vomiting, was tiresome in a few patients, but never prevented adequate treatment. Alteration of taste, similar to that occasionally seen with other drugs such as the alkylating agents, occurred only once and was of no importance. The other gastro-intestinal effects encountered are unlike those seen with most other agents and we relate them to the neurotoxic effects.

*Neurotoxic effects*

Peculiar paraesthesiae, cramps and joint pains were sometimes so severe that they became the limiting factor in the treatment. Minor aches and pains were very common but were usually ignored by the patients, unless specifically enquired after. Increasing the dose led to more pain, and there was a direct relationship between dose level and degree of pain in each individual. Colicky abdominal pains were associated with abdominal distension and sometimes constipation: one patient developed extreme distension and was admitted as an emergency, for investigation, to another hospital without our knowledge. A possible association between his symptoms and previous treatment was, unfortunately, not considered and a laparotomy was performed for intestinal obstruction. At operation the whole bowel was grossly distended, so much so that the abdominal cavity was closed with considerable difficulty, but the patient made a rapid, uneventful recovery.

*Alopecia*

This complication occurred in 9 patients, but in none was it complete. Hair grew again rapidly on stopping the agent, and resumption of treatment did not interfere with this process in 7 patients. One patient continued to lose hair until the dose was reduced to 10 mg. weekly. Only one patient was left with sparsely growing hair and he had previously lost all his scalp hair following a course of cyclophosphamide 8 months previously.

*General effects*

Vague general symptoms were not uncommon, maximal 36–48 hours after each injection and rarely lasting for longer than 2–3 days. A few patients found these symptoms limited their activities during this period of time, but usually they were referred to by the patient only if specifically questioned.

### *Tumour Response*

*Hodgkin's disease*

With most oncolytic agents, more satisfactory responses are seen in Hodgkin's disease than in any other condition, and vinblastine is no exception. All patients treated had advanced disease with generalised lymph node enlargement, and sometimes invasion of extra-lymphatic structures. A good response was usually obvious within 1–2 weeks of starting treatment. Fluctuations in the natural course of the disease make it impossible to attribute an improvement of less than 4 weeks to any specific treatment, and an improvement was therefore recorded as such only if it continued steadily for at least this period of time. A " good " response in this condition indicates general improvement with disappearance of enlarged nodes and spleen, and a moderate response indicates general improvement, with a reduction in size of nodes or spleen to under half their original size. Remissions of 1–20 months have occurred, the longest to date being 20 months, the patient continuing in remission. The average period of good objective control was 7 months.

A good remission occurred more frequently when the Hodgkin's disease was known to have responded previously to other agents, and a response was much less likely when the tumour had proved refractory to treatment with other drugs (Table IV). This limits the usefulness of vinblastine, but nevertheless some remissions followed failure to control the disease by other means. In addition, vinblastine is particularly indicated when haematopoietic depression makes the administration of many other agents extremely hazardous. In general, we regard vinblastine as the most effective and least toxic of the available agents for the control of systemic Hodgkin's disease.

TABLE IV.—*Response to Vinblastine after Previous Administration of Other Agents*

| Response to previous chemotherapeutic agents | Subsequent response to vinblastine |
|---|---|
| Good—13 patients . . | None—6 patients |
| | Good—7 patients |
| None—10 patients . . | None—7 patients |
| | Good—3 patients |

*Other lymphomas*

Patients with lymphosarcoma and reticulum sarcoma sometimes respond well to vinblastine, but less frequently than with Hodgkin's disease (Table I). Our results so far suggest that the drug of choice in the management of these lymphomas is still an alkylating agent, chlorambucil being the most generally satisfactory and least toxic. But it is well worth trying vinblastine if more conventional drugs are not controlling the disease.

*Non-lymphomatous tumours*

Various other types of cancer were treated with vinblastine, with little success. One bronchial carcinoma decreased in size for a period of 6 weeks and one malignant thymoma regressed for a similar period of time.

Generalised breast cancer has been reported to respond to vinblastine (Armstrong, Dyke and Fouts, 1962) but in our experience the results have been very disappointing. Two patients showed a marked response, but the final result was unsatisfactory. One required 20 mg. weekly to control the disease and this dose level could not be maintained because of haematopoietic depression. The second patient had extensive tumour involving both breasts and spreading over the whole of the chest wall. There was a remarkable improvement for 12 weeks, when the patient lapsed from treatment. Within 3 weeks the tumour spread rapidly and it now proved resistant to large doses of vinblastine.

### DISCUSSION

Vinblastine given by intermittent intravenous injection is undoubtedly a highly effective drug in the management of various generalised malignant lymphomas, and at present it appears to us to be the treatment of choice in the control of advanced, Stage III, Hodgkin's disease (Jelliffe and Thomson, 1952).

Various toxic effects occur, some quite commonly, but on the whole the drug is remarkably free from unpleasant side effects. In our experience, all oncolytic agents at present in clinical use have various tiresome side effects, some of which may not be related by the patient to that drug, until a particular course of treatment has stopped. Vinblastine is no exception and many patients refer later to vague symptoms which they had noticed, but which had not distressed them during the period of its administration. Most patients notice a sense of general well-being and this more than makes up for vague aches and pains which they may have.

The beneficial effects of vinblastine may occur with extreme rapidity. To illustrate this, we refer to one patient with Stage III Hodgkin's disease which had proved refractory to various alkylating agents. Treatment was started after the patient had been restricted to his bedroom for 4 months. Two days after the first injection, he dressed and went actively about the house in spite of painful joints and muscle cramps, and he noticed the disappearance of the irritation and sweating which had been a regular nightly feature. Four days after starting treatment his wife telephoned to see if he could be allowed to carry out minor tasks in the garden.

Such a dramatic effect is certainly not seen in all patients, and it may be as long as 3–4 weeks before the benefits become obvious, particularly with tumours which do not originate in lymphatic tissue. Nevertheless, the rapid benefits which may follow its use permit a favourable comparison of vinblastine with chloroethylimine as an agent for the treatment of urgent problems in the management of Hodgkin's disease. Vinblastine can also be most satisfactory in the long term control of this disease, and it is particularly useful when there is already evidence of depression of the haematopoietic system.

The greatest practical disadvantages of vinblastine are the need for regular intravenous injections, and the extremely painful reactions which inevitably follow any extravasation of the drug. There are patients who have no superficial veins of any reasonable size and rarely this has forced us to abandon the use of vinblastine.

This report refers essentially to the use of vinblastine sulphate by intermittent intravenous injection in the management of generalised malignant disease. When given by intra-arterial infusion, a very powerful cancerocidal effect can be produced in the area supplied by that artery and this use of vinblastine will be referred to elsewhere. When given systemically the oncolytic effect is, of course very much less marked. It is important to emphasise that vinblastine, and all other oncolytic drugs given systemically, are totally unsatisfactory substitutes for energetic radiotherapy in the attempted radical treatment of localised malignant lymphomas and it is clear that surgery remains the most hopeful method of treatment in the attempted cure of relatively radio-resistant, operable tumours arising elsewhere.

### SUMMARY

Vinblastine sulphate is an extremely active oncolytic agent. Tumour control can be obtained with only minor side effects. The toxic effects of this drug suggest that its mode of action is very different from other previously reported agents. The most striking effects are produced in Hodgkin's disease, in the treatment of which it may be the present drug of choice, but regression of other tumours can be produced. Further long-term study of the drug is indicated before its real value can be decided.

As with all other known oncolytic agents, the systemic administration of vinblastine sulphate is not a substitute for radiotherapy or surgery in the radical treatment of localised tumours.

We wish to thank the many consultants who referred patients for treatment and the various members of the medical and nursing staff who helped in the management of these patients.

We are most grateful to Messrs. Eli Lilly and Company Limited for generous supplies of vinblastine, and for their help during this investigation.

Part of the expenses of the study were defrayed by the British Empire Cancer Campaign for Research.

### REFERENCES

Armstrong, J. G., Dyke, R. W. and Fouts, P. J.—(1962) *Cancer Chemother. Rep.*, **18**, 49.

Hodes, M. E., Robin, R. J. and Bond, W. H.—(1960) *Cancer Res.*, **20**, 1041.

Jelliffe, A. M. and Thomson, A. D.—(1952) *Brit. J. Cancer*, **9**, 21.

Warwick, O. H., Darte, J. M. M. and Brown, T. C.—(1960) *Cancer Res.*, **20**. 1032.